# The Mythology of Insect-Loving Japan

**DOI:** 10.3390/insects13030234

**Published:** 2022-02-26

**Authors:** Hideto Hoshina

**Affiliations:** Faculty of Education, Bunkyo Campus, University of Fukui, Fukui 910-8507, Japan; hhoshina@f-edu.u-fukui.ac.jp; Tel.: +81-776-27-8692

**Keywords:** insect, mythology, Kojiki, Nihonshoki, Fudoki

## Abstract

**Simple Summary:**

This article discusses the inclusion of insects in Japanese myths. It is believed that the Japanese have more cultural affinity towards insects when compared to elsewhere in the world. It is reasonable to expect that this attitude toward insects is based on the prevalence of insects in Japanese myths. However, insects appear only seven times in the texts that were reviewed in this study, and are only twice presented in a positive light. Gods and emperors in Japanese lore are not commonly depicted as speaking to insects, or as instructing them to work. Neither are gods, nor humans, portrayed as taking the form of insects. In conclusion, relationships between gods, emperors, and insects are sparse in Japanese myths. In terms of cultural entomology, modern Japanese attitudes toward insects, and the origin thereof, require further investigation.

**Abstract:**

Japanese people are perceived to have a relatively more favorable disposition towards insects than individuals from other nations. Given that insects frequently appear in myths from all over the world, I researched Japanese mythology as a potential origin of this positive outlook toward insects. I reviewed the ancient records Kojiki, Nihonshoki, and Fudoki, and found seven cases where insects appear. In all cases, the insects played relatively minor roles. They did not speak, nor were they under the command of gods or emperors. They did not feature as main characters in ancient poetry, and gods/emperors did not take the shape of any insects. In only two instances were insects featured in a positive light. In general, relationships between gods, emperors, and insects are weak in Japanese mythology, and hence mythology does not appear to be the primary source of Japanese affinity for insects.

## 1. Introduction

It is a commonly held belief that Japanese people have a relatively more favorable disposition towards insects than people from other parts of the world [1,2]. A Greek-Irish writer, who was naturalized as a Japanese citizen, admired the Japanese sentiment for insects and regarded it as completely different from the attitude of European people [3]. An American chemistry teacher, and guest of the Japanese Government, marveled at Japanese girls having insect cages with fireflies [4,5]. A French academic, who was a professor at Gifu Keizai University, pointed out that the stability of the Japanese pet-insect market for children is unique and extraordinary, given the wide availability of animations, comics, and television games for children in Japan [6]. It is also believed that cultural sensibilities and feelings for insects reflect the role played by insects in mythology [7].

Insects frequently appear in myths all over the world and often play important roles in the stories [8,9]. Kritsky and Cherry [10] divided the roles of insects in tales into the following three categories: (1) positive (beneficial, industrious, or helpful), (2) negative (evil, plague-forming, pestilent, or harassive), and (3) others which do not fit into the other two categories [10]. For example, in Greek mythology, bees brought honey to the young Zeus [11]. In ancient Egypt, scarab beetles were believed to be the force that moved the sun across the sky [12,13]. Moreover, scarab beetles were associated with the self-created God Atem, who was associated with resurrection and new life [14]. The ancient Greeks and Romans adopted, to varying degrees, the sacred Egyptian scarab and regarded it as a good luck charm [15]. Therefore, the bees and scarab beetles were given a positive role. Conversely, in a Samoan myth, Nifoloa, a monstrous insect, has a long, sharp tooth, and preys on lone people walking home [16]. In a North American Tlingit legend, one man killed an evil giant and burned its body, and each particle of ash became a mosquito [17]. Naturally, Nifoloa and the mosquitoes play a negative role. In a Chinese myth, a chamberlain’s wife committed suicide after the tragic death of her husband, and her clothes changed into numerous butterflies [18]. According to a myth of the Cochiti in the southwestern United States, the *Eleodes* beetle (Tenebrionidae) does a handstand, and hides its face in the dirt, because it is ashamed of having done a poor job while placing stars in the sky [19]. The butterflies and the *Eleodes* beetle are depicted as playing neither a positive nor a negative role.

Accounts of Japanese mythology can be found in ancient texts such as Kojiki, Nihonshoki and Fudoki. Kojiki and Nihonshoki contain accounts of the creation of the universe, stories of many gods, and the histories of the ancient Imperial Family. These are the oldest Japanese history books, compiled in the early eighth century by the government. Furthermore, ancient regional legends, products, and climate information were recorded in a series of books known as Fudoki. Fudoki were edited in the eighth century by the *Kokushi*, who correspond to the present prefectural governors under the orders of the central government. Unfortunately, most Fudoki have been lost; only some parts, such as Izumo Fudoki and Harima Fudoki, have been handed down to the present day (Izumo and Harima are names of places, and correspond to the present day Shimane and Hyogo Prefectures, respectively).

With this work, I aim to uncover the roles of insects in Japanese mythology, and to consider whether the perceived Japanese affinity for insects is a reflection of those roles. Japanese language is very difficult to read, and as a result, very little is commonly known about the role of insects in Japanese myth.

## 2. Methods

The word “myth” is commonly used, but dictionaries provide several different definitions. Differences between myth and religion are often unclear [10]. Therefore, it is challenging to define myths precisely. In this report, I designate the following stories as Japanese myth: the creation of the universe according to the Japanese, tales of many gods, histories from the first Jinmu Emperor to the 25th Buretsu Emperor written in Kojiki and Nihonshoki, and ancient regional legends in Fudoki. Japanese historians accept that the existence of all the emperors from the 1st to the 25th is not conclusively proven. For example, the existence of the 10th Sujin Emperor, who has been regarded as an important founding Emperor, is not proven [20]. Furthermore, not all histories of the first to 25th Emperors set out in Kojiki and Nihonshoki are factual, and many legends and tales are interwoven with them. Therefore, for the purpose of this study, I treated histories of the first to 25th Emperors as a part of Japanese mythology.

To report the instances of insects appearing in Japanese myths, I examined Kojiki, Nihonshoki, and Fudoki.

## 3. Results

I found seven cases of insects appearing in Japanese myths.

1. God Wakumusuhi is a son of the God of fire, Kagutsuchi. Silkworms and mulberries arose from the top of Wakumusuhi’s head (Nihonshoki).
2. Goddess Ôgetsuhimeno-kami conjured delicious food items out of her nose, mouth, and anus, and served them to many gods. However, God Susanoo-mikoto thought that she had sullied the food and killed her. Just then, silkworms arose from her head (Kojiki).
3. God Susanoo-mikoto tried to spite his son-in-law, the god Ôkuninushino-kami. One day he commanded Ôkuninushino-kami to eliminate lice from his hair. Ôkuninushino-kami examined his head, as he was ordered, but found that instead of lice, there were centipedes among the hairs. Susanoo-mikoto also ordered Ôkuninushino-kami to sleep in a room filled with centipedes and wasps, but Ôkuninushino-kami could sleep deeply, because his wife, the goddess Suseribimeno-mikoto, had given him a magical insect-repellent cloth in advance (Kojiki).
4. God Hoakarino-mikoto had a violent temper. His father, God Ônamuchino-mikoto, disliked his son, left him, and escaped by ship. Hoakarino-mikoto was furious with his father, and he conjured up a huge windstorm. Many silkworms that were caught by the windstorm fell onto a hill. Thereafter, the hill was named Himejiga-oka (Harima Fudoki). In Japanese, *hime* and *oka* mean a silkworm and a hill, respectively. Incidentally, the name, *Himeji*, is still used today, and a World Heritage Site Himeji Castle is located in Himeji City, Hyogo Prefecture.
5. The first Jinmu Emperor mounted a hill after assuming the throne at 31 years old and looked towards his country. He said, “I have a wonderful country. It is narrow but surrounded by mountains like a pair of dragonflies copulating”. As a result, his country came to be known as *Akitsu-shima* (Nihonshoki). In Japanese, *akitsu* and *shima* mean a dragonfly and a land, or an island, respectively. The correct interpretation of his remark is difficult, and there are various opinions about his intent. According to one account, in that scene, the copulation of dragonflies represents the fecundity of a rice paddy [21]. Therefore, the summary of his intent is perhaps that he had acquired a rich country.
6. Iwanohimeno-mikoto, the Empress of the 16th Nintoku Emperor, was jealous and ran away from home because her husband kept a concubine. To mediate between the couple, three retainers falsely reported to the Emperor that the Empress just went to a house to watch silkworms. Nintoku Emperor also visited the house, watched silkworms with the Empress, and reconciled with her (Kojiki).
7. The 21st Yûryaku Emperor went on a hunt. A biting fly bit him on an unnamed field. Just then, a dragonfly came, grabbed the biting fly in its mouth, and flew away. The Emperor was highly pleased and named the field *Akitsuno* (Kojiki and Nihonshoki). In Japanese, *akitsu* and *no* mean a dragonfly and a field, respectively.

## 4. Discussion

The booming Japanese pet insect market supports the notion that Japanese people have a strong affinity for insects. In 2005, approximately 190 million foreign rhinoceros beetles and stag beetles were imported into Japan [22], and in 2006, the market size of stag beetles alone was considered to be over USD 100 million [23]. Having said that, I could find only seven cases where insects appear in surviving Japanese myths; and, in the context of Kritsky and Cherry’s [10] classification of insects’ roles in myths, only twice were insects (dragonflies) featured in a positive light. Silkworms featured three times in a neutral light, and lice and wasps featured once in a negative light.

Moreover, Japanese insects are not shown to speak to or change into gods/emperors. According to Kojiki, there are two instances where animals (a mouse and a toad) speak to God Ôkuninushino-kami. First, a mouse appeared and advised him to escape to a hole underground when he was surrounded by fires in a field. Second, a toad came forward and suggested, “God Kuebiko must know his name”. When God Ôkuninushino-kami was approached by a strange god (whose name he did not know) aboard a ship at the shore of Izumo. Thus, mammals and amphibians are shown as speaking to gods in Japanese myth, but insects seemingly cannot talk to gods or emperors at all.

In Nihonshoki, a tragic hero, Prince Yamato-takeruno-mikoto, who could not ascend the throne, transformed into a white bird immediately after his death. In addition, in *Tango* Fudoki, a turtle changed into a beautiful girl. She was from a pure land away from the world (Tango is included in the present Kyoto Prefecture). Thus, in Japanese mythology, gods and humans are seen transforming into birds and non-avian reptiles, and vice versa. However, there are no scenes in which insects turn into humans or vice versa.

Furthermore, the roles of insects in Japanese myth are less important than those of vertebrates. According to Kojiki, the goddess Amaterasu-ôkami dispatched a crow to the first Jinmu Emperor to guide him on the road. He adopted the crow and used it as a messenger to advise his enemy to surrender. Thus, in Japanese myth, birds are shown as working under gods and emperors. Although the dragonfly was shown hunting a biting fly for the 21st Yûryaku Emperor, the dragonfly helped him without being ordered to do so. According to the historic records, Japanese gods and emperors did not command insects to perform tasks.

The relationship between insects and gods or humans is minimal in Japanese myth, and the roles played by insects in the stories appear to be fairly minor. Despite this, there are some instances where modern Japanese affinity for insects may be linked to myths, as may be the case for dragonflies. Since ancient times, the Japanese have felt an affinity towards dragonflies and have regarded them as insects that bring victory [24]. Dragonflies feature in two events with heroic emperors in Kojiki and Nihonshoki. The first Jinmu Emperor is a heroic founder of the Empire and Yûryaku Emperor is more than just the 21st Emperor; he is also a hero who is described as a great military leader [25].

In summary, the Japanese’s fondness of insects does not appear to stem primarily from the Japanese myths examined in this study. The inclusion of other historic texts in a study of this nature, for e.g., religious texts, may reveal more examples of insects in Japanese mythology, since the lines between history, legend, and religion are often blurred.

## 5. Conclusions

Both foreign and local perceptions, together with the highly valued pet insect market, suggest that Japanese people have a relatively higher affinity for insects than people of other nationalities. It is possible that the perceived Japanese affinity for insects is exaggerated, and I would recommend this as a subject for evaluation, together with an in-depth study of the origins of the modern Japanese affinity for insects. I could find only seven cases where insects appear in Japanese myths, and the roles that insects play in the myths appear relatively insignificant in many cases. Therefore, the relationship between insects and gods or humans is minimal in Japanese mythology, and it is unlikely that Japanese mythology is at the root of the perceived Japanese affinity for insects.

## Data Availability

No new data were created or analyzed in this study. Data sharing is not applicable to this article.
